# CK19 is a sensitive marker for yolk sac tumours of the testis

**DOI:** 10.1186/s13000-015-0243-y

**Published:** 2015-03-24

**Authors:** Felix Bremmer, Philipp Ströbel, Hubertus Jarry, Jasmin Strecker, Nadine Gaisa, Arne Strauß, Stefan Schweyer, Heinz-Joachim Radzun, Carl-Ludwig Behnes

**Affiliations:** Institute of Pathology, University Medical Center, University of Göttingen, Göttingen, Germany; University Medical Center, University of Göttingen, Göttingen, Germany; Institute of Pathology, RWTH Aachen University, Aachen, Germany; Department of Urology, University Medical Center, University of Göttingen, Göttingen, Germany; Gemeinschaftspraxis Pathologie, Starnberg, Germany

**Keywords:** Testicular germ cell tumours, Yolk sac tumours, CK19, Immunohistochemical expression

## Abstract

**Background:**

Malignant germ cell tumours are the most common malignant tumours in young men. They are histologically divided into seminomas and non-seminomas. Non-seminomas are further subdivided into embryonic carcinomas, yolk sac tumours, chorionic carcinomas, and teratomas. For the therapeutic management it is essential to differentiate between these histological subtypes.

**Methods:**

Investigated cases included normal testis (n = 50), intratubular germ cell neoplasia (n = 25), seminomas (n = 67), embryonic carcinomas (n = 56), yolk sac tumours (n = 29), chorionic carcinomas (n = 2), teratomas (n = 7) and four metastases of YST’s for their CK19 expression. In addition Leydig cell- (n = 10) and Sertoli cell- tumours (n = 4) were included in this study.

**Results:**

All investigated seminomas, embryonic carcinomas as well as normal testis and intratubular germ cell neoplasias did not express CK19. In contrast, all investigated yolk sac tumours strongly expressed CK19 protein. These findings became also evident in mixed germ cell tumours consisting of embryonic carcinomas and yolk sac tumours, although CK19-expression could also be observed in analysed chorionic carcinomas and epithelial components of teratomas.

**Conclusion:**

CK19 proved to be a sensitive marker to identify yolk sac tumours of the testis and to distinguish them from other germ cell tumours, especially seminomas and embryonic carcinomas.

**Virtual slides:**

The virtual slide(s) for this article can be found here: http://www.diagnosticpathology.diagnomx.eu/vs/4075546891400979.

## Background

Testicular germ cell tumours (TGCTs) are the most common malignancies in young men at the age of 15 to 40 years [1]. The incidence has constantly increased over the last 40 years [2]. TGCTs are histologically and clinically grouped into seminomas (SEM) and non-seminomas. Non-seminomas are further subdivided into embryonic carcinomas (EC), yolk sac tumours (YST), chorionic carcinomas (CC), and teratomas (TER) [3].

The distinction between the different histological tumour types is important for the therapeutic management. Several immunohistochemical markers have been established to differentiate these tumour types. Intratubular germ cell neoplasia unclassified (IGCNU) and seminomas show an expression of D2-40, CD117, OCT3/4, and SALL4 in most of cases [4-7]. Embryonic carcinomas express OCT3/4, NANOG, CD30, Sox-2, and cytokeratin [4,8,9]. YST express cytokeratin, glypican-3, and alpha-feto protein (AFP), albeit the latter, being represented in only about 50% of all cases [10,11]. In addition to their expression in YST, AFP and Glypican-3 are expressed in hepatic tumours [12,13]. In the context of AFP and Glypican-3 CK19 is used as a cell marker of the hepatic biliary tract [14] and its progenitor cells [15].

In addition to intrahepatic cholangiocarcinoma [16] and hepatocellular carcinoma [17] several other tumours have been shown to express CK19, including papillary thyroid carcinoma [18], breast cancer [19], lung cancer [20].

New biomarkers, which are useful for the diagnosis of YST are rare. In this study we will introduce CK19 as a new relevant marker for YST within the group of TGCT.

## Methods

### Tissue samples of primary TGCT

Tumour tissues from orchiectomy specimens were acquired from the University Medical Centre Göttingen and RWTH Aachen, Germany. Tumours were classified and staged on the basis of the WHO classification [21]. The investigated cases included tumour-free testis (n = 50) and IGCNU (n = 25) from patients with TGCT’s. Tumour samples implied pure seminomas (n = 57), pure embryonic carcinomas (n = 20), pure YST (n = 2), pure CC (n = 1) and mixed germ cell tumours (n = 38) consisting of SEM + EC (n = 10), EC + YST (n = 18), EC + YST + TER (n = 7), SEM + YST (n = 2) and EC + CC (n = 1). Furthermore, four metastases of YST in the omentum majus (n = 1), lung (n = 1) and lymph nodes (n = 2) have been investigated. In addition 14 tumours of the sex cord/stroma group were also investigated, in particular Leydig cell tumours (n = 10) and Sertoli cell tumours (n = 4). All specimens were immediately fixed in formalin and embedded in paraffin. Ethical approval for using human material in the present study was obtained from the ethics committee of the University Medical Centre Göttingen and RWTH Aachen. Two independent investigators evaluated all tissue sections.

### Immunohistochemistry

Immunohistochemical reactions were performed on 4-*μ*m formalin-fixed and paraffin-embedded tissue sections. Sections were stained on a Dako Autostainer with the Dako EnVision™ FLEX+ detection system (Dako, Glostrup, Denmark). The system detects primary mouse and rabbit antibodies and the reaction is visualised by EnVision™ FLEX DAB+ Chromogen. Using EnVision™ FLEX+ Mouse (LINKER) or EnVision™ FLEX+ Rabbit (LINKER) (Code K8019) signal amplification of primary mouse antibodies or primary rabbit antibodies were presented, respectively.

The deparaffinization, rehydration, and heat-induced epitope-retrieval (HIER) were carried out in one step with the 3-in-1 procedure buffer (Dako Target Retrieval Solution), pH 9 high ((10x)(3-in-1) Code S2375)) or pH 6 low, [(10x)(3-in-1) Code S1699)] at 97°C using a PT Link, Pre-Treatment Module (Dako). Tissue samples were analysed by light microscopy after 8 minutes counterstaining with Meyer’s haematoxylin (Dako). As primary antibodies we used Anti-CK19, −Glypican-3 and -AFP (Table 1).

**Table 1:** **Antibodies used in this study**

**Antibody**	**Clonality**	**Buffer**	**Dilution**	**Incubation**	
Anti-CK19	monoclonal mouse	high	Ready to use	20 min	(IR 615 Dako)
Anti-AFP	polyclonal rabbit	high	Ready to use	30 min	(IR 6500 Dako)
Anti-Glypican-3	monoclonal mouse	low	Ready to use	30 min	(G1829R06 DCS Immunoline)

## Results

### CK19 expression in normal testis, IGCNU, seminoma, and embryonic carcinoma

**Figure 1:**
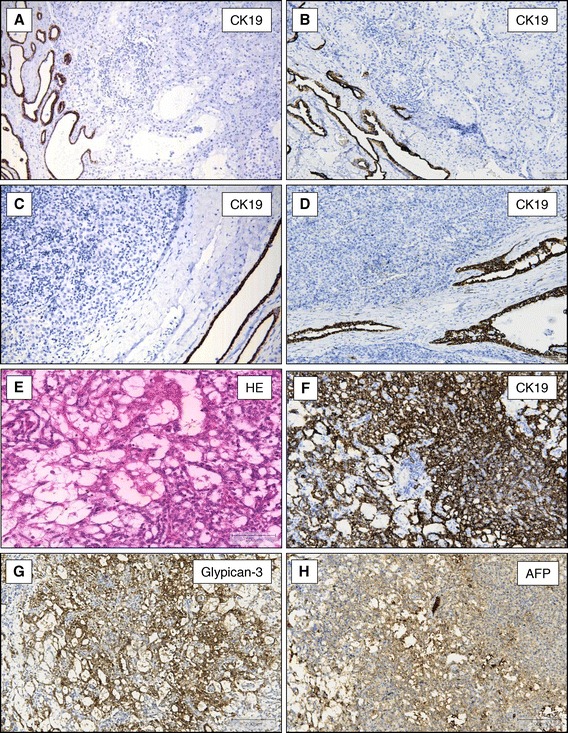
**CK19-expression in normal testis and germ cell tumours of the testis.** CK19 is not expressed in normal testis (**A**, x40), IGCNU (**B**, x40), seminoma (**C**, x100), and embryonic carcinoma (**D**, x100). In all cases the rete testis showed a strong cytoplasmic and membrane bound expression of CK19 **(A-D)**. YST of the testis with microcystic pattern (**E**, HE staining x200) express CK19 (**F**, x100), Glypican-3 (**G**, x100), and AFP (**H**, x100).

Normal testis, cells of the interstitium and Leydig cells did not express CK19 (Figure 1A), Glypican-3, or AFP. In addition IGCNU (Figure 1B), all examined seminomas (Figure 1C) and embryonic carcinomas (Figure 1D) did not express CK19, Glypican-3, or AFP. In all examined tissues the rete testis and the epidymides showed a strong cytoplasmatic and membrane bound expression of CK19 protein (Figure 1 A-D).

### CK19 expression in YST, teratomas, and chorionic carcinomas

**Figure 2:**
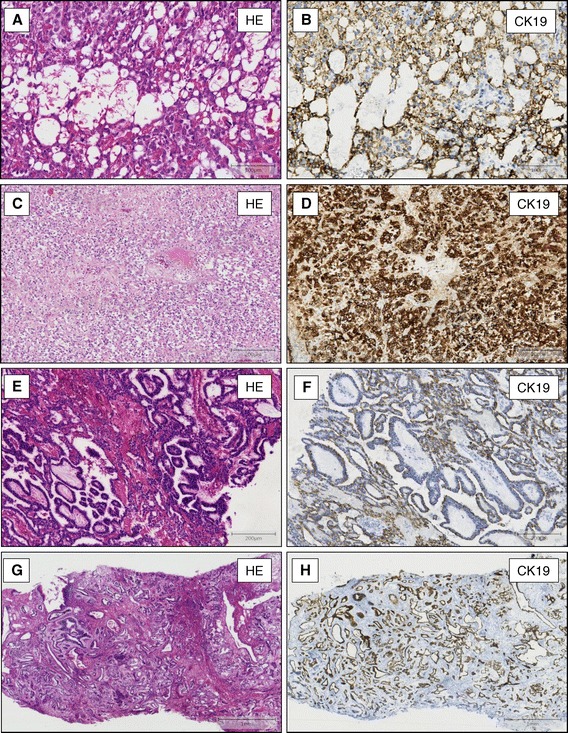
**CK19-expression in different growth pattern of yolk sac tumours.** CK19 shows a strong cytoplasmic and membrane bound expression in YST with microcystic- (**A**, HE staining x200 and **B**, immunostaining, x200), solid- (**C**, HE staining x100+ **D**, immunostaining, x100), papillary- (**E**, HE staining x100 and **F**, immunostaining, x100) and endodermal sinus- rowth pattern (**G**, HE staining x40 and **H**, immunostaining, x40).

**Figure 3:**
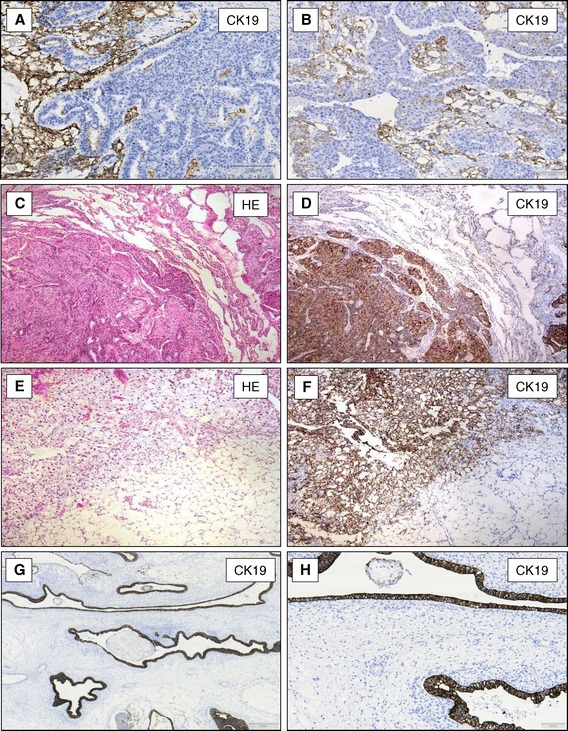
**CK19-expression in mixed germ cell tumours of the testis and metastases.** YST showing a strong expression of CK19 and embryonic carcinoma component in the same tumour does not express CK19 protein (**A** and **B**, x100). Omentum majus with metastasis of an YST (**C**, HE staining x40) expressing CK19 protein (**D**, x40). Pulmonary metastases of an YST of the testis (**E**, HE staining x40) express CK19 (**F**, x40). Intestinal mucosa in teratomas (**G**, x40+ **H**, x100) shows positive CK19-expression.

All 29 YST strongly expressed Glypican-3 and CK19. The immunohistochemical signals were located within the cytoplasma and at the cell membrane (Figure 1 E-G). In contrast, only 18 of these tumours showed a patchy expression of AFP (Figure 1H). CK19 expression was evident in the different growth patterns of YST such as reticular/microcystic pattern (Figure 2A and B), solid pattern (Figure 2C and D), papillary pattern (Figure 2E and F) or endodermal sinus pattern with festooned appearance (Figure 2G and H). The CK19 positivity could also be shown in tumours consisting of YST and embryonic carcinomas (Figure 3A and B) as well as in metastasis of the omentum majus (Figure 3C and D), lung (Figure 3E and F) and lymph nodes (data not shown).

Teratomas, in line with their histologic heterogeneity, showed a great diversity of CK19 expression, which was mainly confined to the different epithelial structures, whereas mesodermal derivatives did not show CK19 expression (Figure 3G and H). Both investigated chorionic carcinomas showed a strong CK19 expression (data not shown).

### CK19 expression in sex cord/gonadal stroma tumours

The ten Leydig cell tumours and four Sertoli cell tumours of the testis did not express CK19. Table 2 shows the expression pattern of all investigated proteins investigated in this study (Table 2).

**Table 2:** **Expression pattern of CK19, Glypican-3 (GLP-3), and AFP in testicular tumours**

	**TF(n = 50)**	**IGCNU(n = 25)**	**SEM(n = 67)**	**EC(n = 56)**	**YST(n = 29)**	**CC(n = 2)**	**LCT(n = 10)**	**SCT(n = 4)**
**CK19**	0/50	0/25	0/67	0/56	29/29	2/2	0/10	0/4
**GLP-3**	0/50	0/25	0/67	0/56	29/29	0/2	0/10	0/4
**AFP**	0/50	0/25	0/67	0/56	18/29	0/2	n.d	n.d

(TF = normal testis, IGCNU = intratubular germ cell neoplasia, SEM = seminoma, EC = embryonic carcinoma, YST = yolk sac tumour, TER = teratoma, CC = chorionic carcinoma, LCT = leydig cell tumours, SCT = sertoli cell tumours).

## Discussion

YST are rare malignant germ cell tumour of the testis. They occur in about 44% of cases in adults as a component of a mixed tumour, whereas pure YST are rare at this age [21,22]. Pure YST are the second most common malignant germ cell tumours of infancy [23,24]. YST show many different growth patterns such as the reticular/microcystic, macrocystic, solid, hepatoid, enteric, glandular/alveolar, papillary, endodermal-sinus-like or myxomatous polyvesikular [25] subtype. Although the distinction of these (several) growth patterns is not essential for therapy, the vast morphological diversity complicates the diagnosis of YST. In this study we looked for a new sensitive marker, which can be used for the differential diagnosis of YST.

The keratin family constitutes the intermediate filament proteins responsible for the structural integrity of epithelial cells and is subdivided into cytokeratins and hair keratins. CK19 is the smallest acidic cytokeratin [26]. CK19 expression has been detected in several types of human cancers, including papillary thyroid carcinoma, breast cancer, lung cancer, intrahepatic cholangiocarcinoma, and HCC [16-20].

All 29 examined YST showed strong CK19 expression. This expression was also found in mixed germ cell tumours consisting of more than one histological type and could be used to distinguish YST from other germ cell tumour components. In 1987 Bartokva et al. described a (focal) CK19 expression in 14 examined embryonic carcinomas [27]. In our study, however, CK19 expression could not be detected in a total number of 56 embryonic carcinomas. This became particularly evident in mixed tumours consisting of YST and embryonic carcinomas. Furthermore, CK19 expression was also confirmed in metastases of an YST in the omentum majus, lung, lymph nodes, and in a primary YST of the ovary. Glypican-3 as an established marker for YST could also be observed in all analysed cases of YST, whereas AFP showed an expression in only 18 out of 29 cases.

We found CK19 expression in the two analysed chorionic carcinomas as described also by Clark et al. [28]. All other analysed cases of malignant and non-malignant tumours of the testis did not show CK19 expression. Because glypican-3 can be also expressed in chorionic carcinoma, this tumour has to be excluded by morphological criteria and expression of β-HCG [10,11].

For the diagnosis of YST several markers such as Keratin, Glypican-3 or in some cases AFP can be used. CK19 proves to be an additional useful positive marker which can be used together with these established markers, especially Glypican-3.

It has been shown previously that cells of hepatocellular carcinomas expressing e.g. CK19 may result from dedifferentiation of hepatocytes into a progenitor cell/biliary phenotype [29,30]. According to the diagnostic role of CK19 in yolk sack tumours as we described in this study, CK19 expression might be a clue for differentiation to a hepatocyte or biliary phenotype in this tumour entity. This would be in line with the known expression of glypican-3 or AFP in these tumours.

CK19 is widely applied as a post-operative diagnostic marker of papillary thyroid carcinoma. It is mandatory to elucidate further whether this holds true also for YST.

## Conclusion

CK19 proved to be a sensitive marker for the diagnosis of a primary YST of the testis and metastases. Its expression can be used to differentiate YST of the testis from other germ cell tumours and sex cord/gonadal stroma tumours. In addition, CK19 may be a possible and helpful pre/post-operative serum marker of YST.
